# GRANDPARENTAL CAREGIVING AND CO-RESIDENCE, SOCIOECONOMIC STATUS, AND MORTALITY IN RHODE ISLAND CITIES AND TOWNS

**DOI:** 10.1093/geroni/igz038.1166

**Published:** 2019-11-08

**Authors:** Julia Broccoli, Steven A Cohen, Mary L Greaney

**Affiliations:** University of Rhode Island, Kingston, Rhode Island, United States

## Abstract

Grandparents co-residing with their grandchildren is becoming increasingly more common, with over 1.5 million grandchildren living with their grandchildren in the U.S. Furthermore, the number of grandparents who are primary caregivers for their grandchildren has also increased, which can negatively effects the grandparents’ physical and mental health, and increase social isolation and financial burden. However, the associations between grandparental caregiving and health outcomes are not well understood on a population level. Therefore, the purpose of this study was to assess associations between grandparental caregiving, socioeconomic status, and population health outcomes. Using mortality data (2009-2011) from the Rhode Island (RI) Department of Health and life table methods for each RI city/town, life expectancy at age 65 (LE65) and age-standardized mortality rates (ASMR) were calculated and linked to data from the American Community Survey on grandparental caregiving responsibilities, grandparental living arrangements (co-residence), poverty status, and demographics. Correlations and multivariable linear regression modeling were used to evaluate associations among LE65, ASMR, grandparental caregiving and co-residence, demographics, and poverty. Both LE65 (rho=-0.382, p=0.016) and ASMR (rho=0.327, p=0.042) were associated with the percent of grandparents living with grandchildren. The percent of grandparents as primary caregivers to their grandchildren was not significantly associated with LE65 or ASMR. ASMR was associated with the percent of grandparents living in poverty (rho=0.401, p=0.013) and overall poverty (rho=0.363, p=0.023). These results highlight conditions of community-based living and role of primary caregivers at an older age that should be further explored to improve the health of grandparents, particularly in multi-generational homes.

